# Presumptive myocarditis with ST-Elevation myocardial infarction presentation in young males as a new syndrome. Clinical significance and long term follow up

**DOI:** 10.1186/1476-7120-9-1

**Published:** 2011-01-18

**Authors:** Marcello Costantini, Giuseppe Oreto, Alberto Albanese, Anna Ranieri, Giovanni De Fabrizio, iovanni Sticchi, Antonio Lauretti, Sergio Capone, Cristina Tritto, Claudio Fachechi, Realino Renna, Antonio Montinaro, Eugenio Picano

**Affiliations:** 1 Struttura Complessa di Cardiologia, Ospedale Santa Caterina Novella, Galatina, Italy; 2 Dipartimento di Medicina e Farmacologia, Università di Messina, Messina, Italy; 3 Istituto di Fisiologia Clinica del CNR -Sezioni di Lecce e Pisa, Italy

## Abstract

**Background:**

Acute myocarditis may mimic myocardial infarction, since affected patients complain of "typical" chest pain, the ECG changes are identical to those observed in acute coronary syndromes, and serum markers are increased. We describe a case series of presumptive myocarditis with ST segment elevation on admission ECG.

**Methods and Results:**

From 1998 to 2009, 21 patients (20 males; age 17-42 years) were admitted with chest pain, persistent ST segment elevation, serum enzyme and troponine release. All but one patients had fever and flu-like symptoms prior to admission. No abnormal Q wave appeared in any ECG tracing, and angiography did not show significant coronary artery disease. Patients remained asymptomatic at long term follow-up, except 2 who experienced a late relapse, with the same clinical, electrocardiographic and serum findings as in the first clinical presentation.

**Conclusion:**

Presumptive myocarditis of possible viral origin characterized by ST elevation mimicking myocardial infarction, good short term prognosis and some risk for recurrence is relatively frequent in young males and appears as a distinct clinical condition.

## Background

Myocarditis may occasionally mimic acute myocardial infarction, with a similar clinical presentation characterized by chest pain, ECG changes consistent with acute coronary syndromes, and serum markers increment [1-3]. We have previously reported, in a cohort of 11 young male patients, a clinical condition consistent with myocarditis and characterized by ST segment elevation, serum markers release and good short term outcome [4]. In the present study we describe our updated experience with a larger case series (21 patients) and an extended follow up.

## Methods

From January 1^st^, 1998 to December 31th 2009, 21 patients with the following clinical/ECG pattern were admitted to the Coronary Care Unit of a Southern Italian small town (Galatina). All patients were young; all but one was males; the coronary risk profile was low: 11 were light smokers (less than 10 cigarettes/day) and no one suffered from hypertension or diabetes. Hospital admission was needed for prolonged chest pain with ECG changes (ST elevation > 1 mm in at least two leads) and serum markers increment.

In all patients ECG, cardiac enzymes assay and rest echocardiogram was obtained daily. In 8 patients, RNA-enterovirus search was performed by PCR-method on stool and saliva specimens. Coronary angiography was performed in all patients during the acute phase (in 3 cases at hospital admission). In 5 patients in whom the admission diagnosis was STEMI, thrombolysis with r-TPA was performed; in these, fibrinogen and D-dimer levels were evaluated before and after treatment. The remaining patients received only aspirin, associated with heparin in 9.

After discharge, all patients were followed-up for 65.6 ± 49.1 months (range 1-130), with clinical evaluation, ECG, echocardiogram and exercise stress-test. No medication was indicated at hospital discharge.

The study was approved by our institutional review committee. All patients gave informed consent for inclusion in the study.

## Results

**Figure 1:**
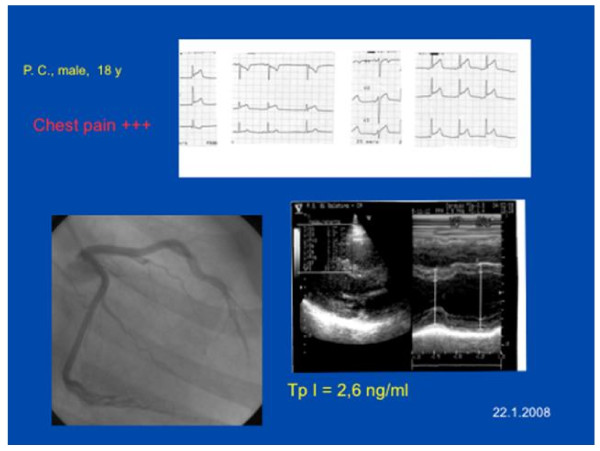
**A typical sample case (case 21). See text**.

Figure 1 shows a characteristic pattern of presentation in our population. The typical sample case is represented by a young man presenting with typical chest pain, normal global LV function, and ECG modification consistent with acute myocardial ischemia, normal coronary arteries and abnormal levels of serum markers of myocardial damage. The main clinical, laboratory and electrocardiographic findings are listed in table 1. All patients were young (mean age 27.9 ± 7.02 years, range 17-42), 20 were males, no one was a heavy smoker but 11 were light smokers. All patients but one had a recent history of flu-like episode, with fever and diarrhoea. The only young female showed a hairy chest.

**Table 1:** Clinical, laboratory and electrocardiographic findings

Patient, sex, age (years)	Admission month	Recent Fever	CK-MB, U/L admission/peak (Troponine I, ng/ml)	Coronary risk factors (smoke)	ECG leads with ST elevation on admission ECG	Abnormal T waves on discharge ECG	Follow up (months)	Relapse
**16) 1 SW, M, 19**	**March**	**+**	**71/73 (NA)**	**0**	**I, aVL, V4-V6**	**Diphasic V4-V6**	**130**	**no**

**2 TG, M, 24**	**November**	**+**	**39/83 (NA)**	**0**	**I, aVL, V6**	**Negative I, II, aVL, V5,V6**	**122**	**yes**

**3 CE, M, 32**	**December**	-	**76/77 (NA)**	**0**	**I, II, V4-V6**	**Negative V4-V6**	**121**	**no**

**4 AA, M, 38**	**February**	**+**	**67/120 (NA)**	**1 (smoke)**	**II, III, aVF, V5, V6**	**Diphasic III, aVF**	**119**	**no**

**5 CM, M, 30**	**April**	**+**	**91/130 (NA)**	**1 (smoke)**	**II, III, aVF**	**Negative III,V6**	**105**	**no**

**6 BM, M, 17**	**January**	**+**	**60/60 (NA)**	**0**	**I, II, aVF, V4-V6**	**Negative II, III, aVF, V4-V6**	**96**	**no**

**7 CR, M, 32**	**August**	**+**	**24/43 (17)**	**1 (smoke)**	**II, III, aVF,V4-V6**	**None**	**77**	**no**

**8 CG, M, 39**	**October**	**+**	**17/30 (16)**	**1 (smoke)**	**I, aVL**	**Negative I, aVL, V6**	**63**	**no**

**9 SS, M, 28**	**December**	**+**	**23/23 (10.5)**	**1 (smoke)**	**II, III, aVF, V5-V6**	**None**	**61**	**no**

**10 CR, M, 21**	**April**	**+**	**34/34 (5.4)**	**1 (smoke)**	**II, III, aVF, V6**	**None**	**57**	**no**

**11 CR, M, 28**	**April**	**+**	**17/17 (3.6)**	**0**	**I, aVL**	**Flat I, aVL**	**57**	**no**

**12 SM, M, 26**	**July**	**+**	**8/14 (4.3)**	**0**	**II, III, aVF**	**Negative II, III, aVF**	**54**	**yes**

**13 CA, M, 19**	**April**	**+**	**21/21 (16.1)**	**0**	**II, V6**	**None**	**45**	**no**

**14 LF, M, 42**	**October**	**+**	**10/12 (5.3)**	**0**	**I, II, III, aVF, V6**	**Negative III, V6**	**38**	**no**

**15 AG, M, 20**	**April**	**+**	**55/55 (29)**	**1 (smoke)**	**I, aVL, V2-V6**	**Negative V4-V6**	**38**	**no**

**16 DA, M, 33**	**March**	**+**	**7/16 (2)**	**1 (smoke)**	**II, III, aVF, V5-V6**	**Flat II**	**34**	**no**

**17 DM, F, 31**	**April**	**+**	**19/19 (9.4)**	**1 (smoke)**	**II, III, aVF**	**Negative III**	**33**	**no**

**18 IA, M, 25**	**February**	**+**	**30/30 (3.4)**	**0**	**II, III, aVF**	**Negative II, III, aVF**	**23**	**no**

**19 FR, M, 35**	**December**	**+**	**14/14 (1.9)**	**1 (smoke)**	**II, III,, aVF**	**None**	**13**	**no**

**20 AM, M, 27**	**January**	**+**	**19/24 (2,3)**	**0**	**II, III, aVF**	**Negative II,III, aVF**	**1**	**no**

**21 PF, M. 18**	**January**	**+**	**23/28 (3,56)**	**1 (smoke)**	**I, aVL, V2-V6**	**Negative I, II, aVL, V2-V6**	**1**	**no**

NA = not available

+ = present

- = absent

**Figure 2:**
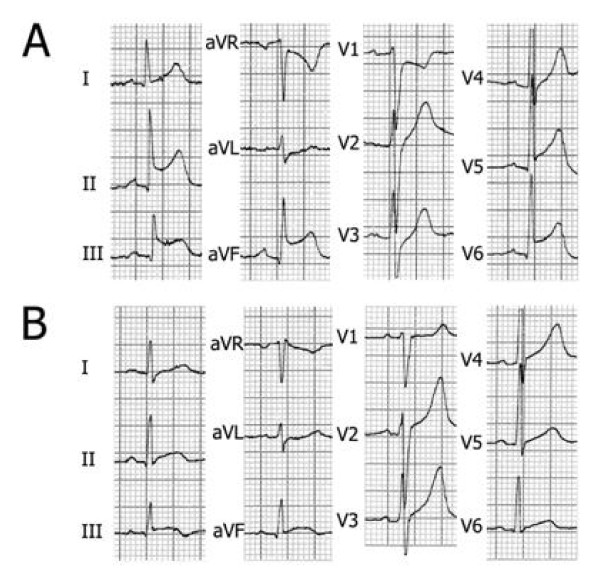
**Electrocardiograms recorded on admission (A) and discharge (B) from patient n. 4**.

All patients were admitted for acute chest pain that had started 50-425 minutes before admission. The characteristics of chest pain were always typical for acute coronary syndrome: midsternal constrictive pain, often radiating to the left arm and/or to the neck. Physical examination was unremarkable; in particular, no pericardial friction rub was heard and no signs of heart failure were evident. The admission ECG showed upward concave ST segment elevation in all cases (inferior in 5, lateral in 5, infero-lateral in 10, anterior in 1), followed, in the subsequent days, by T wave inversion; no abnormal Q waves, however, appeared in any patient. Figure 2 reports a typical example of ECG evolution (case 4).

Cardiac serum enzymes, as well as troponin, were abnormal, but with an atypical pattern, being increased on admission (even when the elapsed time from chest pain onset was very short), with a relatively low peak, and late normalization (5-8 days). At echocardiography, no wall motion abnormality was detected in 17 patients, whereas a transient regional dysfunction, lasting only a few days, was observed in 4 cases, with good correspondence between the echocardiographic location of wall motion impairment and the ECG leads showing ST segment and/or T wave abnormality. Ejection fraction (Simpson's rule) was normal in all patients (mean 67 ± 11%).

In 5 patients (n. 2, 4, 5, 10, 11), an initial diagnosis of STEMI prompted thrombolytic treatment with r-tPA. In 4 of these, fall of serum fibrinogen levels, not associated to any D-dimer increase, was observed. Coronary angiography showed normal coronary arteries in all cases, including the subset (n = 3) who underwent the exam immediately after hospital admission. Screening for enterovirus, performed in 8 patients, was positive in 5.

The clinical course was uneventful in 19 cases. A transient third sound was heard in one patient, without any other sign or symptom of heart failure. In one case, frequent asymptomatic ventricular arrhythmias (polymorphic ventricular ectopic beats and runs of non sustained ventricular tachycardia) occurred during the first 2 days. All patients were discharged in good clinical state, 8.5 ± 2.8 days after admission.

At follow-up, 19/21 cases remained asymptomatic, with normal physical findings, ECG, echocardiogram, and exercise stress-test. In two cases (2 and 12), a relapse was observed, 89 and 45 months after the first episode. In both patients, the recurrence of the clinical condition was preceded by a flu-like disease, with fever and diarrhoea, with same clinical presentation and ECG and serum modifications as in the first episode. Coronary angiography was repeated in one of these patients and did not show significant abnormalities.

## Discussion

Our patients suffered from an acute non-coronary cardiac disease characterized by chest pain, ST segment elevation, enzyme and troponin release, normal coronary angiogram, good prognosis and relative risk of late relapse (10%). This acute condition affected mostly young males. Several feature are likely to exclude an ischemic origin: a) young age and low coronary risk profile; b) atypical ECG evolution, with slow ST segment normalization and no Q wave appearance; c) atypical pattern of bio-markers release, with high admission value even when hospital admission occurred within 1 hour from pain onset, myocardial involving before symptoms appereance; d) absence of D-dimer level elevation in spite of marked fibrinogen decrease following treatment with r-TPA, an observation suggesting the likelihood that no intravascular thrombi were present; e) normal coronary angiogram.

It is likely that the clinical condition may be of viral origin: a) all but one patients experienced fever with flu-like symptoms, often associated with diarrhoea, a few days before admission; b) the disease shows a seasonal prevalence (maximal incidence in winter and spring, with peak in April), parallel to that of flu-like viral infections in our region; c) the evidence of enterovirus infection in 5 out the 8 cases in which PCR research was performed.

An intriguing finding is that all but one of our patients was of male gender. According to the small sample size, this could be a casual phenomenon. Alternatively, it could represent the clinical counterpart of an experimental observation pointing out that some viral strains induce myocarditis only in adult male animals [5]. Coxackievirus B3 infection results in cardiac inflammation in male, but not in female B1.Tg.Ealpha mice [6]. In addition, although weanling male BALB/c mice show minimal cardiac injury after infection, young adult male animals develop severe myocarditis while female age-matched mice do not [7]. Moreover, female animals given testosterone demonstrate in their hearts ten times higher virus levels than female animals given estradiol [8]. Testosterone could affect susceptibility to infection by increasing virus receptor expression on endothelial cells and myocites, and/or by influencing the immune-response [9,10]. It should be pointed out that the only young woman in our population had a hairy chest, a phenomenon that could suggest a possible role of testosterone in favouring a presumptive viral myocarditis. Interestingly enough, the disease can relapse, aven after many years, with the same clinical/electrocardiographic presentation. The characteristics of the clinical relapse pose a viral etiology of the disease, with a likely immune pathogenesis [11]. Finally, a warning should be recommended to physicians before treating patients with atypical clinical presentation for myocardial infarction. There are a few features that physicians should check in order to avoid potential potential life-threatening side effects of thrombolysis: young age; male sex; low coronary risk profile; concomitant flu-like symptoms in the few days before admission; atypical electrocardiographic presentation; atypical serum markers release. In such a clinical scenario, coronary angiography is preferable to thrombolysis.

## Study Limitations

The diagnosis of presumptive myocarditis was empiric and deductive, based on a very likely (and in some cases proved) recent viral infection, clinical presentation and evolution, ECG pattern, cardiac bio-markers release, and lack of epicardial coronary artery disease. Myocardial biopsy could have resulted in unquestionable demonstration of myocarditis [12], but was not performed because not strictly appropriate in this clinical context [13].

## Conclusions

STEMI-like presumptive myocarditis occurring in young males appears as a distinct acute cardiac non-coronary illness whose origin is not unequivocally proved, although viral aetiology is suggested. The disease shares some characteristics with other conditions that mimic acute coronary syndromes but are associated with angiographically normal coronary arteries. In this group, tako-tsubo disease has recently become very popular, and several "variants" have been described, with more or less pronounced differences from the original report [14]. Tako-tsubo syndrome, however, is more common in females over 45 years, is often preceded by a psychological stress, and is associated with wall motion abnormalities mainly affecting the apical region [15]. The herewith discussed disease, in contrast, is characterized by male gender prevalence, young age of patients, preceding flu-like disease, and absence of any stereotyped ventricular wall motion involvement (table 2). Further studies, involving larger cohorts of patients, are necessary to define whether STEMI-like presumptive myocarditis affecting young males is indeed a syndrome deserving further attention.

**Table 2:** Parallelism between "Galatina's" and tako tsubo like syndromes

Syndrome	Galatina's	Tako-tsubo like
STEMI-like presentation	yes	yes

Usually benign	yes	yes

Occasionally recurrence	yes	yes

Gender predominance	male	female

Age (years)	< 40	> 45

Prodomal phase	Flu-like	Psycological stress

Favourite target region	inferior	apical

## Authors' contributions

MC conceived of the study, and participated in its design and coordination. AA, AR, GDF, GS, AL, SC, CT, CF, RR, AM participated in the design of the study and performed the statistical analysis. GO and EP critically revised the manuscript and participated in study design. All authors read and approved the final manuscript.

## Competing interests

The authors declare that they have no competing interests.

## References

[B1] Narula J Khaw BA Dec GW Palacios IF Southern JF Fallon JT Recognition of Acute Myocarditis Masquerading as Acute Myocardial Infarction New Engl J Med 1993 328 100 104 10.1056/NEJM199301143280205 8416421

[B2] Sarda L Colin P Boccara F Daou D Lebtahi R Faraggi M Myocarditis in patients with clinical presentation of myocardial infarction and normal coronary angiograms J Am Coll Cardiol 2001 37 786 792 10.1016/S0735-1097(00)01201-8 11693753

[B3] Magnani JW Dec GW Myocarditis: current trends in diagnosis and treatment Circulation 2006 113 876 890 10.1161/CIRCULATIONAHA.105.584532 16476862

[B4] Costantini M Tritto C Licci E Sticchi G Capone S Montinaro A Myocarditis with ST-Elevation Myocardial Infarction presentation in young man. A case series of 11 patients Int J Cardiol 2005 101 157 158 10.1016/j.ijcard.2004.01.023 15860403

[B5] Lyden D Olszewski J Huber S Variation in susceptibility of Balb/c mice to coxsackievirus group B type 3-induced myocarditis with age Cell Immunol 1987 105 332 339 10.1016/0008-8749(87)90081-5 3494528

[B6] Huber SA Kupperman J Newell MK Hormonal Regulation of CD4+ T-cell Responses in Coxsackievirus B3-Induced Myocarditis in Mice J Virol 1999 73 4689 4695 10233928 10.1128/jvi.73.6.4689-4695.1999 PMC112510

[B7] Lyden D Olszewski J Huber SA Influences of Sex Hormones on Coxsackievirus Group B, Type 3 Induced Myocarditis in BALB/c Mice Eur Heart J 1987 8 Suppl J 389 391

[B8] Lyden DC Olszewiski J Feran M Job LP Huber SA Coxsackievirus B-3-Induced Myocarditis. Effect of Sex Steroids on Viremia and Infectivity of Cardiocytes Am J Pathol 1987 126 432 438 3030117 PMC1899641

[B9] Frisancho-Kiss S Nyland JF Davis SE Frisancho JA Barret MA Rose NR Sex differences in coxsackievirus B3-induced myocarditis: IL-12Rβ1 signaling and IFN-γ increase inflammation in males independent from STA T4 Brain Res 2006 1126 139 147 10.1016/j.brainres.2006.08.003 16949558

[B10] Huber SA Increased susceptibility of male BALB/c mice to coxsackievirus B3-induced myocarditis: role for CD1d Med Microbiol Immunol 2005 194 121 127 10.1007/s00430-004-0221-6 15107990

[B11] Merkler D Horvath E Bruck W Zinkernagel RM de la Torre JC Pinschewer DD "Viral déjà vu" elicits organ-specific immune disease independent of reactivity to self J Clin Invest 2006 116 1254 1263 10.1172/JCI27372 16604192 PMC1430358

[B12] Caforio AL Calabrese F Angelini A Tona F Vinci A Bottaro S A prospective study of biopsy-proven myocarditis: prognostic relevance of clinical and aetiopathogenetic features at diagnosis Eur Heart J 2007 28 1326 1333 10.1093/eurheartj/ehm076 17493945

[B13] Cooper LT Baughman KL Feldaman AM Frustaci A Jessup M Kuhl U Levin GN Narula J Starling RC Towbin J Virmani R Heart Failure Society of America and Heart Failure Association of the European Society of Cardiology. The Role of Endomyocardial Biopsy in the Management of Cardiovascular Disease. A Scientific Statement From the American Heart Association, the American College of Cardiology and the European Society of Cardiology Circulation 2007 116 2216 2233 10.1161/CIRCULATIONAHA.107.186093 17959655

[B14] Buchholz S Rudan G Tako-tsubo syndrome on the rise: a review of the current literature Postgrad Med J 2007 83 261 264 10.1136/pgmj.2006.052423 17403953 PMC2600040

[B15] Bybee KA Kara T Prasad A Lerman A Barsness GW Wright RS Systematic review: transient left ventricular apical ballooning: a syndrome that mimics ST-segment elevation myocardial infarction Ann Int Med 2004 141 858 865 15583228 10.7326/0003-4819-141-11-200412070-00010

